# Time‐Division Multiplexing for Parallel Transmission at Ultra‐High Field With Limited RF Channels

**DOI:** 10.1002/mrm.70230

**Published:** 2025-12-19

**Authors:** Felix Glang, Georgiy A. Solomakha, Dario Bosch, Klaus Scheffler, Nikolai I. Avdievich

**Affiliations:** ^1^ Magnetic Resonance Center Max Planck Institute for Biological Cybernetics Tübingen Germany; ^2^ Institute of Biomedical Imaging Graz University of Technology Graz Austria; ^3^ Department of Biomedical Magnetic Resonance Eberhard Karls University Tübingen Tübingen Germany; ^4^ MRI Core Facility of the Medical Faculty University of Tübingen Tübingen Germany

**Keywords:** flip angle homogeneity, high‐field MRI, multiplexing, RF switch, SAR

## Abstract

**Purpose:**

Investigating time‐division multiplexing for parallel transmission in ultra high‐field imaging, striving for homogeneous whole brain excitation with a limited number of RF channels.

**Methods:**

A fast RF switch was built to alternately route 8 transmit channels to each row of a double‐row 16‐element transmit coil array at a 9.4 T human MRI system. Methods for SAR monitoring and pulse design for this temporal degree of freedom were developed and investigated in electromagnetic simulations and in vivo measurements, employing parallel transmission kT points pulses aiming for homogeneous whole‐brain excitation. The achievable trade‐off between local SAR and excitation homogeneity was compared for multiplexed and simultaneous transmission.

**Results:**

Using time‐division multiplexing, similar excitation fidelity as with 16 transmit channels can be achieved with only 8 channels. For instance, multiplexing reduces the flip angle inhomogeneity by 2.22‐fold compared to exciting only a single row of the array, and by 1.85‐fold compared to statically splitting and routing 8 channels to 16 transmit coil elements. As a trade‐off, compared to simultaneous excitation, multiplexing requires either increased pulse duration or amplitudes, the latter causing increased SAR. However, with appropriate SAR‐aware pulse design, the multiplexing‐induced local SAR increase can be controlled. This allows for viable pulse design solutions for the considered low‐flip‐angle imaging scenarios.

**Conclusion:**

Time‐division multiplexing allows driving a larger number of transmit elements with a smaller number of RF channels, resulting in improved parallel transmission performance. This opens up new possibilities for using advanced multi‐row transmit coil arrays in sites with only 8 RF channels available.

## 1 Introduction

In ultra‐high field (UHF) MRI, the short RF wavelength leads to pronounced flip angle inhomogeneity and increased power deposition, quantified by the specific absorption rate (SAR). These key limitations can be mitigated through parallel transmission (pTx) [1, 2], using arrays of local transmit (Tx) coil elements driven by independent RF power amplifiers (RFPAs). While in theory, a shaped RF pulse played out through a single RFPA combined with appropriate gradient waveforms can excite nearly arbitrary spatial patterns [3], such multidimensional pulses tend to be excessively long and to cause unfeasibly high SAR [4]. To mitigate this issue, transmitting on multiple local Tx coil elements independently and in parallel can shorten the required RF pulse duration and reduce local SAR [1, 2]. It is desirable to increase the number of degrees of freedom for pTx, that is, using more Tx elements, each driven by its own RFPA, because this enables a more favorable trade‐off between excitation fidelity, SAR, and pulse duration [4, 5, 6]. This is due to the improved numerical conditioning of the pulse design problem when using a larger number of spatially complementary transmit field patterns. However, due to the high cost and complexity associated with additional transmit channels, most UHF scanners are typically equipped with 8 Tx channels, with only a few experimental systems offering up to 16 or 32 Tx channels [7]. As theoretical computations and simulations have shown, these systems can only yield a fraction of the ultimately achievable pTx performance permitted by electrodynamics in terms of the trade‐off between excitation homogeneity and SAR [4, 8, 9].

For human head imaging at UHF, a major challenge is achieving homogeneous whole brain excitation with sufficient longitudinal coverage (i.e., along the head‐foot direction). To address this, various multi‐row coil array designs have been proposed for 7 T [10, 11, 12, 13], 9.4 T [14, 15, 16], and 10.5T [17], aiming to provide the necessary degrees of freedom along the head‐foot direction for successful pTx application [18, 19, 20]. Since these arrays have more than 8 Tx elements, using them in pTx mode requires advanced strategies to split and route the lower number of Tx channels across the larger number of elements. Examples of such approaches are the Butler matrix [21, 22], pair‐wise combination [23], or array‐compressed pTx [24, 25].

In the early days of pTx, serial instead of parallel excitation approaches were explored, in which, for example, multiple coils [26] or a rotatable coil [27] were pulsed sequentially to improve excitation homogeneity. These studies recognized that although the RF fields of sequentially pulsed coils are temporally separated and thus do not interfere directly, their vectorial nature is imprinted into the spin magnetization, leading to observable interference effects. Making use of this effect, it has been demonstrated that the two rows of a 16‐element array can be driven with 8 RFPAs [11, 28], by rapidly alternating the RF waveforms between the 8 elements in each row. This time‐division multiplexing approach was successfully applied for static RF shimming in the human brain at 7 T. More recently, a similar concept has been applied for an experimental reconfigurable transmit coil, in which the RF field distribution of coaxial dipole elements could be electronically modulated [29, 30, 31].

In the present work, we revisit this concept at 9.4 T for a 16‐element double‐row coil array and dynamic pTx in form of 3D kT points excitation [32]. The goal is to achieve homogeneous whole brain excitation, which is relevant for applications in the context of high‐resolution anatomical and functional neuroimaging. In particular, we investigate the influence of multiplexing on SAR in comparison to full simultaneous transmission, which has not been considered before. We introduce a pulse design method that accounts explicitly for the SAR constraints associated with time‐division multiplexing, and examine the inherent trade‐offs of this approach in both electromagnetic simulations and in vivo measurements. First preliminary results of this approach were presented at the ISMRM Annual Meeting [33].

## 2 Theory

The multiplexing principle presented here follows from the linearity of the small tip angle approximation [3] describing multi‐channel RF excitation. Ignoring relaxation and off‐resonance effects, the transverse magnetization after such a pulse is approximated as.

(1) $$M_{\mathrm{xy}}\left(r\right)=i\gamma M_{0}\int_{0}^{T}\;\sum_{c=1}^{N_{c}}\beta_{c}\left(r\right)\;p_{c}\left(t\right)\;e^{ik\left(t\right)\cdot r}\;dt$$

with the initial magnetization $M_{0}$ (assumed as 1 here), the pulse duration T, the summation describing the total $B_{1}^{+}$ field created by the $N_{c}$ coils with spatial transmit sensitivities $\beta_{c}\left(r\right)$ (normalized to input voltage) when driven by the waveforms $p_{c}\left(t\right)$, and the excitation k‐space trajectory $k\left(t\right)=-\gamma \int_{t}^{T}g\left(t^{\prime }\right)dt^{\prime }$ defined by the gradient waveforms g. Discretizing time into $N_{t}$ samples of duration Δt yields.

(2) $$M_{\mathrm{xy}}\left(r\right)=i\gamma M_{0}\cdot \Delta t\cdot \sum_{c=1}^{N_{c}}\sum_{\tau =1}^{N_{t}}\beta_{c}\left(r\right)\;p_{\tau ,c}\;e^{ik_{\tau }\cdot r}$$

with the $N_{t}\times N_{c}$ pulse matrix $p_{\tau ,c}=p_{c}\left(t_{\tau }\right)$, the discrete k‐space locations $k_{\tau }=k\left(t_{\tau }\right)$, and $t_{\tau }=\tau \cdot \Delta t$. Due to the double summation in Equation (2), coil indices c and time step indices τ play equivalent roles and can be rearranged: For example, in this approximation, using two channels to apply a pulse to two coils simultaneously yields the same excitation as using a single channel applying the pulse to each coil consecutively. To see this formally, define a reshaped pulse matrix of size $2N_{t}\times \frac{N_{c}}{2}$ as.

(3) $${\tilde{p}}_{\tilde{\tau },\tilde{c}}=\left\{\begin{array}{l} p_{\frac{\tilde{\tau }+1}{2},\tilde{c}}\;\text{if}\;\tilde{\tau }\;\text{is}\;\mathrm{odd}\\ p_{\frac{\tilde{\tau }}{2},\tilde{c}+\frac{N_{c}}{2}}\;\text{if}\;\tilde{\tau }\;\text{is even} \end{array}\;,\;\tilde{\tau }=1,\ldots ,2N_{t}\;;\;\tilde{c}=1,\ldots ,\frac{N_{c}}{2}\;\right.$$

in which the odd time steps $\tilde{\tau }$ contain the waveform samples for the first half of coils ($c=1,\ldots ,\frac{N_{c}}{2}$), and the even times steps the ones for the second half ($c=\frac{N_{c}}{2}+1,\ldots ,N_{c}$). Next, define time‐dependent transmit sensitivities associated with $N_{c}/2$ channels as.

(4) $${\tilde{\beta }}_{\tilde{c}}\left(r,t_{\tilde{\tau }}\right)=\left\{\begin{array}{l} \beta_{\tilde{c}}\left(r\right)\;\text{if}\;\tilde{\tau }\;\text{is}\;\mathrm{odd}\\ \beta_{\tilde{c}+\frac{N_{c}}{2}}\left(r\right)\;\text{if}\;\tilde{\tau }\;\text{is even} \end{array}\right.$$

which alternatingly switch between the first and second half of the original $N_{c}$ sensitivities. Finally, define $2N_{t}$ pair‐wise repeated k‐space locations as.

(5) $${\tilde{k}}_{\tilde{\tau }}=\left\{\begin{array}{l} k_{\frac{\tilde{\tau }+1}{2}}\;\text{if}\;\tilde{\tau }\;\text{is}\;\mathrm{odd}\\ k_{\frac{\tilde{\tau }}{2}}\;\text{if}\;\tilde{\tau }\;\text{is even} \end{array}\right.$$

The definitions in Equations ((3), (4), (5)) describe a time‐division multiplexing situation that leads to the excitation pattern.

(6) $$M_{\mathrm{xy}}\left(r\right)=i\gamma M_{0}\cdot \Delta t\cdot \sum_{\tilde{c}=1}^{N_{c}/2}\;\sum_{\tilde{\tau }=1}^{2N_{t}}{\tilde{\beta }}_{\tilde{c}}\left(r,t_{\tilde{\tau }}\right)\;{\tilde{p}}_{\tilde{\tau },\tilde{c}}\;e^{i{\tilde{k}}_{\tilde{\tau }}\cdot r}$$

which is identical to the case of simultaneous transmission described by Equation (2). Physically, however, Equation (6) describes a pulse with $2N_{t}$ waveform samples, which is thus twice as long as the pulse described by Equation (2). To retain the original pulse duration, the time steps Δt need to be halved and the pulse amplitudes $\tilde{p}$ need to be doubled. Consequently, the instantaneous power in each channel during the pulse grows by a factor of 4. Fortunately, this does not hold for the time‐averaged power: Consider the contribution of a single time step Δt to the average pulse power in the simultaneous transmission case

(7) $${\bar{P}}_{\text{simul}}=\frac{1}{\mathrm{TR}}\sum_{c=1}^{N_{c}}\frac{{\left|p_{c}\right|}^{2}}{Z}\Delta t$$

with the reference impedance Z=50Ω. In the multiplexing case, this corresponds to two time steps of duration Δt/2, contributing the average pulse power.

(8) $${\bar{P}}_{\text{multiplex}}=\frac{1}{\mathrm{TR}}\left(\sum_{c=1}^{N_{c}/2}\frac{{\left|2\cdot p_{c}\right|}^{2}}{Z}\frac{\Delta t}{2}+\sum_{c=N_{c}/2+1}^{N_{c}}\frac{{\left|2\cdot p_{c}\right|}^{2}}{Z}\frac{\Delta t}{2}\right)=2\cdot {\bar{P}}_{\text{simul}}$$

In summary, the multiplexing approach comes at the cost of either doubled pulse duration or doubled average pulse power. The effect of multiplexing on power deposition in the tissue, that is, SAR, depends on the vectorial relation of the electric field components created by the two coil groups. As derived in the Supporting Information, if these components would add up constructively in simultaneous transmission, there is little to no SAR amplification due to multiplexing. In contrast, if the components would interfere destructively in simultaneous transmission, multiplexing causes a considerably higher SAR. On average, as for the average pulse power, SAR for multiplexing will be twice as high as for simultaneous transmission.

The multiplexing formalism outlined above generalizes theoretically to the extreme case of driving all $N_{c}$ coils with just a single transmitter channel, however, at the expense of an $N_{c}$‐fold increase of either pulse duration or average power.

## 3 Methods

### 3.1 Setup

**FIGURE 1:**
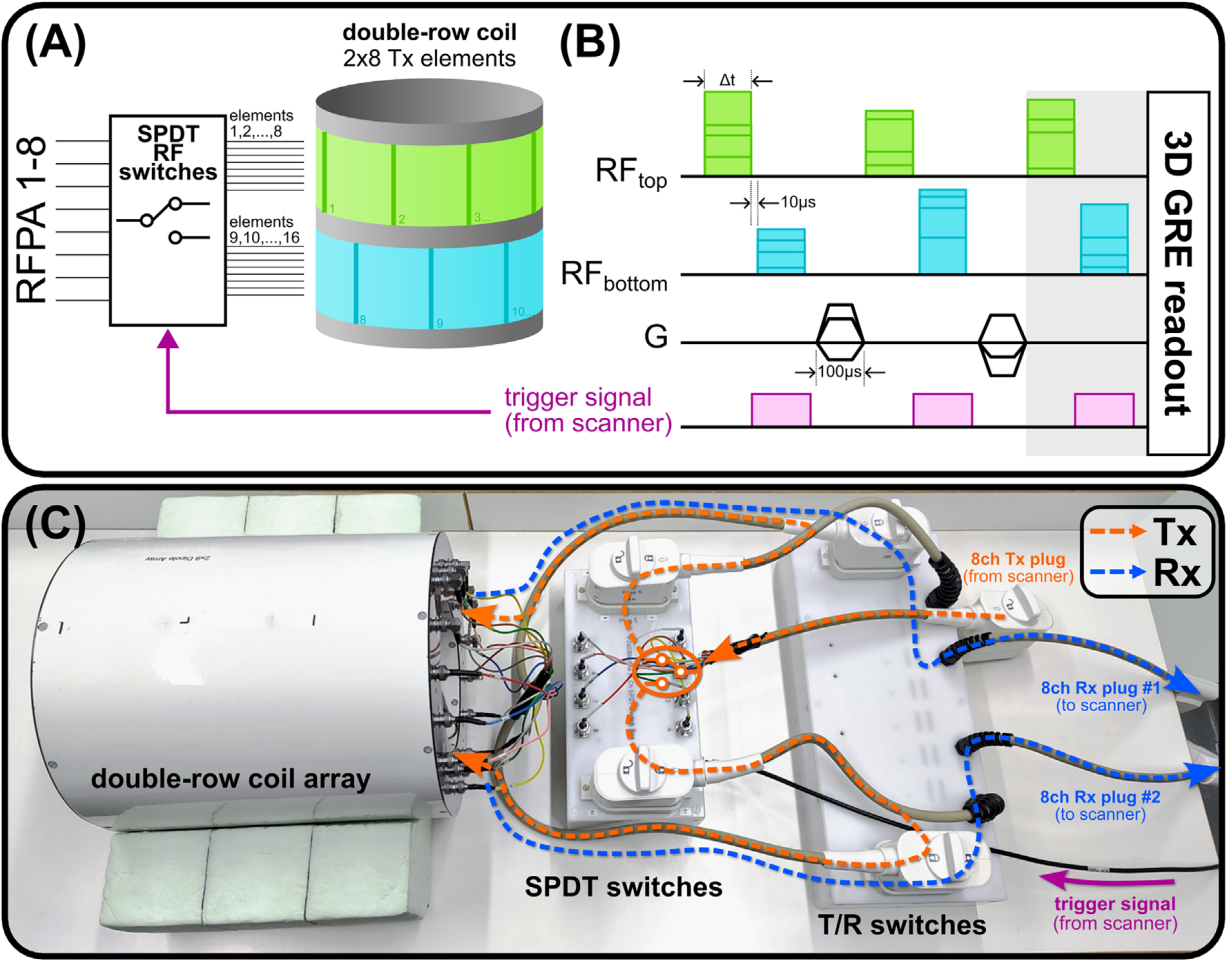
(A) Schematic of the proposed multiplexing setup, which includes 8 high‐power single pole double throw (SPDT) RF switches that are controlled by the trigger output of the scanner to route 8 transmit (Tx) channels alternately to each row of a double‐row 16ch Tx array during the sequence. (B) Sequence diagram of the proposed kT points excitation scheme with RF sub‐pulses switched between the rows. The sub‐pulse duration Δt, switching time (10 μs) and gradient blip duration (100 μs) are indicated accordingly. The RF switches are designed to route to the top row by default and to the bottom row only while the trigger is on. If only the parts in the gray shaded box are played out, this corresponds to the investigated case of multiplexed RF shimming (no gradient blips). (C) Photo of the experimental setup installed outside of the scanner for demonstration purpose. Arrows indicate the transmission (Tx) and reception (Rx) pathways, respectively, as well as the trigger signal.

**FIGURE 2:**
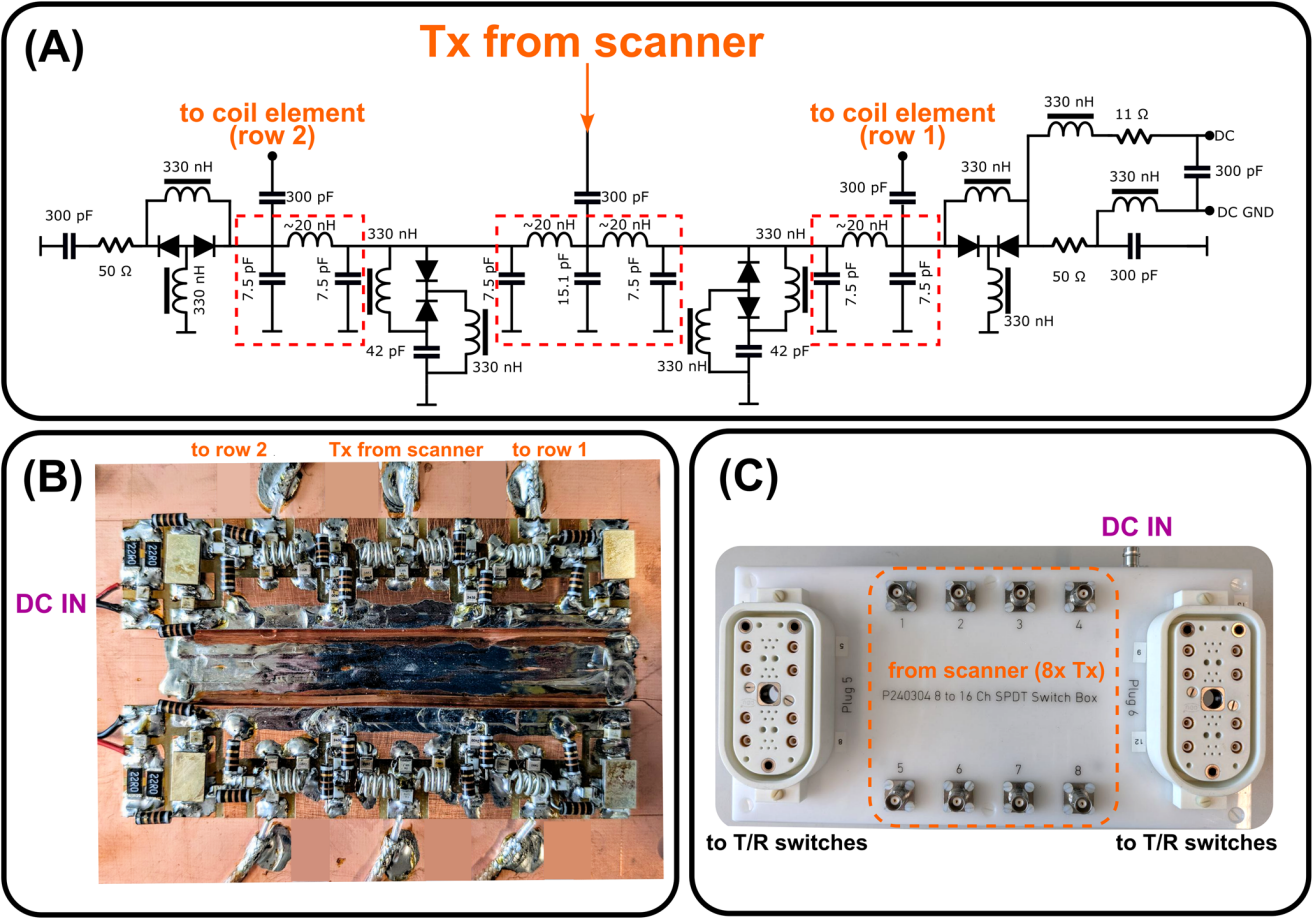
(A) Circuit diagram of an absorptive single‐pole double‐throw (SPDT) RF switch that routes a single transmit (Tx) channel to a coil element from either the top or bottom row of the array. Lumped‐element λ/4 transformers are marked by dashed lines. (B) Photo of the assembled switch electronics. The photo includes two switches, one of which is labeled. (C) Photo of the box containing all RF switches that alternately route eight Tx channels to either row of the array depending on the “DC IN” signal.

The investigations presented here are based on a double‐row 16‐element folded‐end dipole transceiver array developed for human brain imaging at 9.4 T [16]. High‐power absorptive single pole double throw (SPDT) RF switches based on a set of lumped‐element λ/4 transformers and PIN‐diodes (MA4P7102‐1072, MACOM, USA) were constructed to alternately route 8 RFPAs to each row of the array, controlled by the optical trigger output of the scanner during the sequence (Figure 1A). A circuit diagram and photo of the SPDT switch electronics is shown in Figure 2. Each switch was tuned to 400 MHz (^1^H frequency at 9.4 T), but could be relatively easily retuned to another frequency, for example, 300 MHz (7 T) without any modification of the printed circuit board by adjusting the corresponding inductance and capacitor values in the λ/4 transformers (Figure 2). Isolation between the switch channels measured −40 dB or below, and insertion loss per channel was −0.35 dB. The RF switch achieves a switching time of ˜3 μs. Power linearity of the RF switch was verified by performing flip angle mapping in a phantom while increasing the RF input voltage up to the maximum allowed 170 V per channel [34]. Using a separate box with 16 T/R switches [35] connected to the box with 8 SPDT switches as shown in Figure 1C, it was possible to use all 16 dipoles simultaneously during signal reception, but only 8 at a time during transmission.

### 3.2 Simulation and SAR Monitoring

Electromagnetic simulations were performed using CST Studio Suite 2021 (Dassault Systèmes, Vélizy‐Villacoublay, France) on two voxel models [36] (Duke and Ella) to obtain single‐channel *B*_1_^+^ maps, as well as Q‐matrices [37] (2 mm resolution) for the case of driving all 16 elements simultaneously ($Q_{\text{full}}$, $16\times 16\times N_{\text{voxels}}$), only the top row ($Q_{\mathrm{top}}$, $8\times 8\times N_{\text{voxels}}$), or only the bottom row ($Q_{\text{bottom}}$, $8\times 8\times N_{\text{voxels}}$), where $N_{\text{voxels}}$ is the total number of Q‐matrices from Duke and Ella. The CST legacy method was used for 10 g tissue averaging.

**FIGURE 3:**
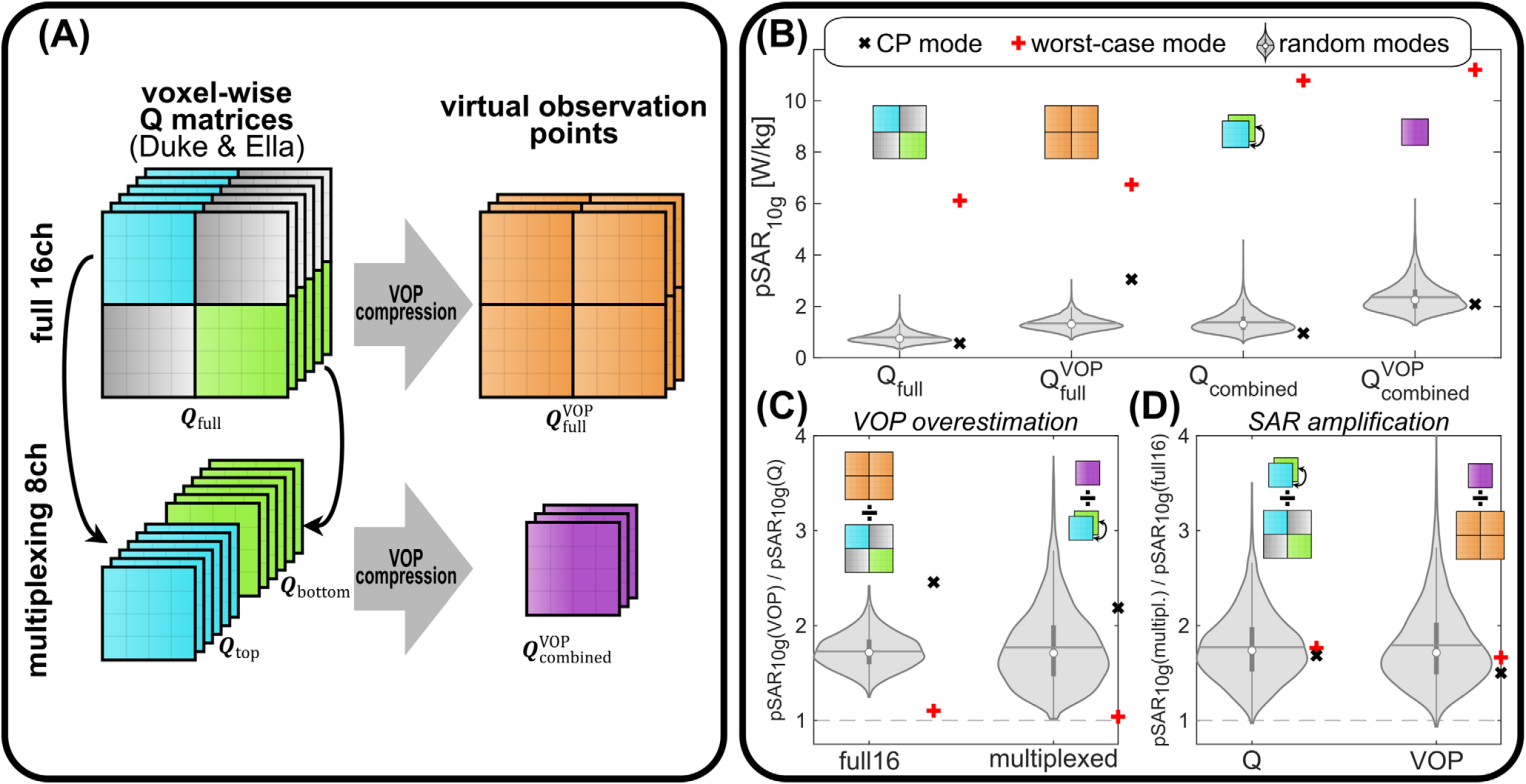
(A) Schematic of the virtual observation point (VOP) compression approach for time‐division multiplexing. For conventional simultaneous 16ch transmission (“full16“), the voxel‐wise 16 × 16 Q‐matrices $Q_{\text{full}}$ are compressed into the VOPs $Q_{\text{full}}^{\mathrm{VOP}}$. In case of multiplexing, the 8 × 8 Q‐matrices corresponding to each row of the array ($Q_{\text{bottom}}$, illustrated as green boxes, and $Q_{\mathrm{top}}$, illustrated as cyan boxes) are concatenated along the voxel dimension and jointly compressed to yield $Q_{\text{combined}}^{\mathrm{VOP}}$. (B) Distributions of pSAR_10g_ values for the 4096 random excitation modes (1 W input power, assuming 100% RF duty cycle) evaluated using the four different sets of SAR‐matrices shown in (A). (C) Distributions of VOP overestimation for simultaneous and multiplexed transmission, i.e., pSAR_10g_ predicted by VOPs divided by pSAR_10g_ predicted by Q‐matrices for the same random modes. (D) Distribution of multiplexing‐incurred SAR amplification for the same modes as obtained from Q and VOP‐matrices. Icons next to the violins indicate the sets of matrices involved in the respective calculation. In addition, results for the CP mode and the worst‐case SAR mode (i.e., the eigenvector corresponding to the largest eigenvalue in $Q_{\text{full}}$) are shown.

With the proposed dynamic switching of 8 Tx channels between the two rows of a 16ch coil, the spatial distribution of the electric field and thus SAR associated with each channel vary in time. This cannot be represented by the SAR monitoring of the scanner system, which requires a single static set of VOP‐matrices [38]. To still ensure safe operation, $Q_{\mathrm{top}}$ and $Q_{\text{bottom}}$ were concatenated along the voxel axis to form $Q_{\text{combined}}$ ($8\times 8\times 2N_{\text{voxels}}$), which was jointly compressed into a single set of VOP‐matrices $Q_{\text{combined}}^{\mathrm{VOP}}$ ($8\times 8\times N_{\mathrm{VOP}}$ with $N_{\mathrm{VOP}}=80$) using the algorithm of Orzada et al. [39] (Figure 3A). This is equivalent to treating the SAR distributions corresponding to the two rows as two different voxel models, both of which must be safely represented by the VOP‐matrices simultaneously. Thus, $Q_{\text{combined}}^{\mathrm{VOP}}$ was generated from four distinct sets of Q‐matrices, two from the Duke and two from the Ella voxel model.

For the 16ch reference case of driving all coil elements simultaneously, VOP compression was applied to $Q_{\text{full}}$, resulting in the VOP‐matrices $Q_{\text{full}}^{\mathrm{VOP}}$ (16×16×80). The worst case overestimation factor according to Orzada et al. [39] was 4.60% for $Q_{\text{full}}^{\mathrm{VOP}}$ and 1.09% for $Q_{\text{combined}}^{\mathrm{VOP}}$. To compare the local SAR predictions based on both Q and VOP‐matrices for both simultaneous and multiplexed transmission, 4096 random 16ch RF modes were drawn and normalized to 1 W input power. The same modes as well as the CP mode and the worst‐case SAR mode were rearranged into 8ch multiplexing shape (Equation (3)) and used to calculate the true physical SAR based on $Q_{\mathrm{top}}$ for the first and $Q_{\text{bottom}}$ for the second sub‐pulse, as well as the prediction of the jointly compressed $Q_{\text{combined}}^{\mathrm{VOP}}$ (assuming doubled amplitudes and halved sub‐pulse durations compared to 16ch simultaneous transmission).

### 3.3 Pulse Design

Pulse design for static RF shimming and kT points excitation was formulated as a magnitude least‐squares optimization [40] with VOP‐based SAR constraints [41] and optional joint optimization of k‐space locations [42] as

$$P^{*},K^{*}=\arg\min_{P,\;K}{\left|\left|\;\left|A\left(K\right)P\right|-b\right|\right|}_{2}^{2}$$

(9) $$s.t.\;\frac{\Delta t}{\mathrm{TR}}\sum_{j=1}^{N_{t}}p_{j}^{H}Q_{n}^{\mathrm{VOP}}p_{j}\leq l{\mathrm{SAR}}_{\max}\;\forall \;n=1,\overset{\ldots }{,}N_{\mathrm{VOP}}$$

with the duration of the RF sub‐pulses Δt, the target flip angle pattern b, the RF pulse amplitudes $p_{j}$ at each time point and channel concatenated as a vector P, the k‐space locations $K=\left\{k_{j},j=1,\ldots ,N_{t}\right\}$ and the maximum local SAR value $l\mathrm{SA}R_{\max}$. Following the spatial domain method [43] and now explicitly considering off‐resonance effects, the system matrix reads

(10) $${\left[A\right]}_{i,\left(\mathrm{jc}\right)}=i\gamma \cdot \Delta t\cdot \beta_{c}\left(r_{i},t_{j}\right)\cdot e^{i\gamma \Delta B_{0}\left(r_{i}\right)\left(t_{j}-T\right)}\cdot e^{ir_{i}\cdot k_{j}}$$

where $\beta_{c}$ is the transmit sensitivity of the c‐th Tx channel and time point $t_{j}$ and voxel location $r_{i}$, $\Delta B_{0}$ the off‐resonance map, and T the total pulse duration. As a generalization of the conventional spatial domain method, this formulation includes the possibility of temporally varying assignment of transmit sensitivities to channels (c.f. Equation (4)). For static RF shimming ($N_{t}=1$), k was fixed to 0 and not optimized. For kT points excitation, the last k‐space location $k_{N_{t}}$ was fixed to 0, while the remaining $k_{j}$ for $j=1,\ldots ,N_{t}-1$ were optimized. In case of multiplexed kT points transmission, two consecutive sub‐pulses share the same k‐space location (c.f. Equation (5) and Figure 1). To incorporate this into the optimization, an optional set of linear equality constraints was added to the optimization problem (Equation (9)), enforcing $k_{2j-1}=k_{2j}\;\forall j=1,\ldots ,N_{t}/2$. Off‐resonance maps $\Delta B_{0}$for the two voxel models were computed numerically based on the known tissue susceptibilities [44] and shimmed up to the second order within the brain tissue mask before including them in the pulse design, leading to a standard deviation of 71 Hz for Duke and 73 Hz for Ella. Solutions to the pulse design problem (Equation (9)) were obtained using the interior‐point method [45] implemented in Matlab (Mathworks, Natick, MA) with user‐supplied analytical Jacobians for the cost and constraint functions (see Supporting Information for details). Data and code for demonstration of the pulse design method can be found at https://github.com/fglang/tdm_ptx.

To compare the performance of different coil driving scenarios such as multiplexed or simultaneous transmission, it is desirable to find the best possible solution for a given local SAR limit $l\mathrm{SA}R_{\max}$. However, since the optimization problem Equation (9) is non‐convex, the interior‐point method can only find local minima, depending on the provided initial values. Thus, the following heuristic procedure was employed to approximate the ideal performance inherent to a specific driving scenario: First, a set of initial k‐space locations $K_{0}$ was drawn from a random uniform distribution in the range $\pm 14\;m^{-1}$ and the initial pulse weights $P_{0}$ were drawn from a complex normal distribution (standard deviation 1 V). Starting from these, an interior‐point optimization without SAR constraints was performed, yielding the result $\left[P_{\infty },K_{\infty }\right]$. Next, a series of 64 RF‐only optimizations were solved with the k‐space locations fixed at $K_{\infty }$, the SAR constraint $l\mathrm{SA}R_{\max}$ linearly decreasing from the initially unconstrained value down to 0.1 W/kg, and P each time initialized with the solutions found in the previous optimization (so‐called “warm start”). In this way, an L‐curve is obtained that shows the trade‐off between excitation fidelity and local SAR constraint [41]. The entire procedure was repeated 1024 times with different random initializations $\left[P_{0},K_{0}\right]$. Finally, the Pareto front [46] of all obtained L‐curves was computed, which approximates the ultimately achievable trade‐off between SAR and excitation fidelity.

Computations with the simulated *B*_1_^+^ maps were performed on a high‐performance computer cluster (28 compute nodes with 64 CPU cores (AMD EPYC 7452) and 512 GB RAM each).

### 3.4 Evaluation

For all investigated cases, the target was a homogeneous excitation of ${\mathrm{FA}}_{\text{targ}}=5{}^{\circ}$ across the brain. Flip angle fidelity of the designed pulses was assessed through the normalized root mean squared error

(11) $$\text{NRMSE}=\frac{\sqrt{\frac{1}{N_{r}}\sum_{i=1}^{N_{r}}{\left(\mathrm{FA}\left(r_{i}\right)-{\mathrm{FA}}_{\text{targ}}\right)}^{2}}}{{\mathrm{FA}}_{\text{targ}}}$$

with the flip angle $\mathrm{FA}\left(r_{i}\right)$ created by the pulse at location $r_{i}$, evaluated across all $N_{r}$ voxels within the brain mask.

The following scenarios were compared:

- “bottom” and “top”: 8ch transmission on a single row only (sub‐pulse duration Δt=0.2ms)
- “full16”: simultaneous 16ch transmission on both rows (Δt=0.2ms)
- “multi8_conv”: multiplexed 8ch transmission, taking the optimized RF pulse from “full16” and converting it without re‐optimization into switched sub‐pulses of halved duration (Δt=0.1ms) according to Equations ((3), (4), (5))
- “multi8_opt”: multiplexed 8ch transmission with dedicated optimization of RF pulses for this scenario (Δt=0.1ms)
- “multi8_2T_opt”: same as “multi8_opt” but without halving the sub‐pulse duration (Δt=0.2ms), leading to a pulse of twice the total duration compared to the previous scenarios
- “passive_split”: 8ch transmission to pairs of adjacent elements from each row with a phase shift of −22.5° between them (corresponding to their geometrical arrangement, which was found to yield the best and most robust performance across the considered scenarios). This can be mathematically described by a 8 × 16 coupling matrix, representing appropriate static power splitters and phase shifters. (Δt=0.2ms)

For all these scenarios, static RF shimming, 2 kT points and 3 kT points excitation was investigated. For the multiplexed scenarios, a 10 μs switching time gap was placed between the sub‐pulses. The gradient blip duration for the kT points was 100 μs (Figure 1B).

### 3.5 Measurements

Using the pulse design methodology discussed in the previous sections and the VOPs generated from electromagnetic simulations, multiplexed and simultaneous pTx were also performed in vivo. Measurements were performed on a Magnetom 9.4 T Plus whole‐body human MRI scanner equipped with 16 Tx channels (Siemens Healthineers, Erlangen, Germany) with a healthy volunteer, after written informed consent and under approval of the local ethics committee. Two sets of experiments were conducted with the 16‐element dipole coil array: (1) Using only 8 of the available Tx channels to drive the coil via the switch, alternating between the top and bottom row of the array, and (2) using all 16 Tx channels for the reference case of driving all elements in both rows simultaneously.

Single‐channel *B*_1_^+^ maps were acquired using a pre‐saturated 3D turboFLASH sequence with interferometric mapping [47, 48, 49] (TR = 2.5 ms, TE = 0.73 ms, GRAPPA 2 × 2, FA = 2°/70°, matrix 64 × 64 × 64, resolution 3.5 mm isotropic). For the 8ch switched setting, *B*_1_^+^ mapping was performed for both states of the switch, that is, rows of the array individually. The single channel maps were spatially smoothed by an isotropic 3D Gaussian kernel (3.5 mm standard deviation) to reduce noise. A brain mask to define the voxels considered during pulse design was obtained using SynthStrip [50]. 3 kT points pulses were optimized according to Equation (9), using the same procedure to obtain L‐curves as described above for the simulations, but with only a single initialization of $\left[P_{0},K_{0}\right]$ set to zero. Computations during the experiments were performed on a local workstation (Intel Xeon W‐2145 3.7 GHz CPU, 8 cores, 128 GB RAM). The obtained pulses were tested using a 3D RF and gradient‐spoiled GRE sequence (TR = 10 ms, TE = 6 ms, BW = 400 Hz/px, GRAPPA 2 × 2, matrix 220 × 220 × 220, resolution 1 mm isotropic) implemented in Pulseq [51] with a custom pTx extension [52] that allows arbitrary RF waveforms for each Tx channel.

## 4 Results

**FIGURE 4:**
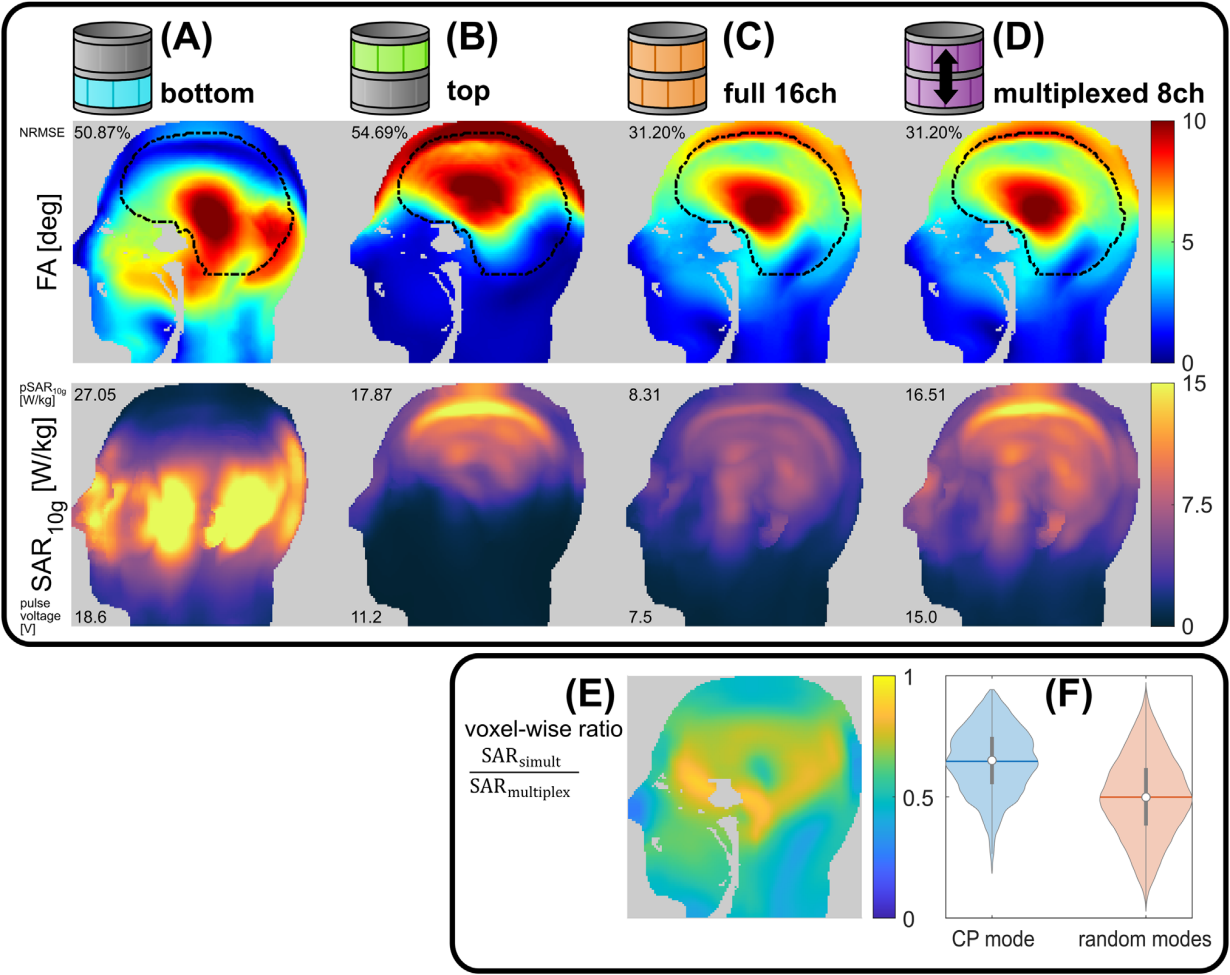
Simulated CP mode excitation for (A, B) 8ch single‐row transmission, (C) simultaneous 16ch transmission using all elements, and (D) time‐division multiplexed 8ch transmission. For each case, the same total pulse duration was assumed and the pulse amplitude was scaled to achieve an average flip angle of 5° in the outlined brain mask. The SAR_10g_ maps are maximum intensity projections across the left–right direction. Numbers indicate the normalized root‐mean‐squared error (NRMSE) with respect to 5° excitation (first row), pSAR_10g_ (second row top, assuming 100% RF duty cycle), and obtained pulse voltages (second row, bottom). (E) Voxel‐wise ratio of the SAR_10g_ maps in (C) and (D), showing the inverse SAR amplification caused by multiplexing (displayed in a single sagittal slice). (F) Violin plots of the same voxel‐wise SAR ratio for the CP mode (left violin, corresponding to the map in (E)), as well as for the same 4096 random RF modes as shown in Figure 3 (right violin). Within the violins, the white dot indicates the median, the horizontal lines the mean, and the gray bars the interquartile range of the respective distribution.

Figure 4 shows simulated flip angle maps and SAR_10g_ maps (obtained from uncompressed Q‐matrices) for a CP mode excitation that was scaled to achieve a mean flip angle of 5°, comparing simultaneous and multiplexed transmission in the Duke voxel model. The corresponding results for the Ella voxel model are shown in Figure S1. As expected, multiplexed 8ch excitation achieves the same flip angle distribution as simultaneous 16ch excitation, however, at approximately twice the 10 g‐averaged peak local SAR (pSAR_10g_), since the total pulse duration was kept constant. In agreement with theory (c.f. Supporting Information, Equation (S4)), the voxel‐wise local SAR ratio of simultaneous and multiplexed transmission varies spatially between 0.18 and 0.94 (Figure 4E), with an average of 0.65 (Figure 4F). This indicates that the electric fields of the two rows predominantly interfere constructively when driven simultaneously in CP mode. This results in less multiplexing‐induced SAR amplification than for randomly selected modes, for which the expected inverse SAR penalty factor is 0.5 (Figure 4F, Equation A5).

Even more relevant than a voxel‐wise comparison of local SAR is the comparison of the respective peak values. Figure 3B compares the pSAR_10g_ values obtained from Q‐ and VOP‐matrices for simultaneous and multiplexed transmission for the 4096 random modes. The median of the pSAR_10g_ distributions is [0.75, 1.30, 1.31, 2.26] W/kg for [$Q_{\text{full}}$, $Q_{\text{full}}^{\mathrm{VOP}}$, $Q_{\text{combined}}$, $Q_{\text{combined}}^{\mathrm{VOP}}$]. The median VOP overestimation (Figure 3C) is similar for “full16” and multiplexed transmission (1.72 vs. 1.71), while the maximum overestimation for “full16” is much lower (2.42 vs. 3.78). The pSAR_10g_ amplification caused by multiplexing (Figure 3D) appears different when observed through Q‐matrices and VOPs, since the latter is additionally scaled by the different overestimation factors for “full16” and multiplexed transmission (Figure 3C). While the median pSAR_10g_ amplification caused by multiplexing appears similar when evaluated by Q‐matrices and VOPs (1.74 vs. 1.71), the maximum amplification observed through the VOPs is additionally inflated compared to the Q‐matrices (4.32 vs. 3.50). Note that in Figure 3D there are some cases, where pSAR_10g_ for multiplexing is slightly lower than for “full16”. This can be explained by spatially varying “hot spots” during multiplexing as described in the Supporting Information.

**FIGURE 5:**
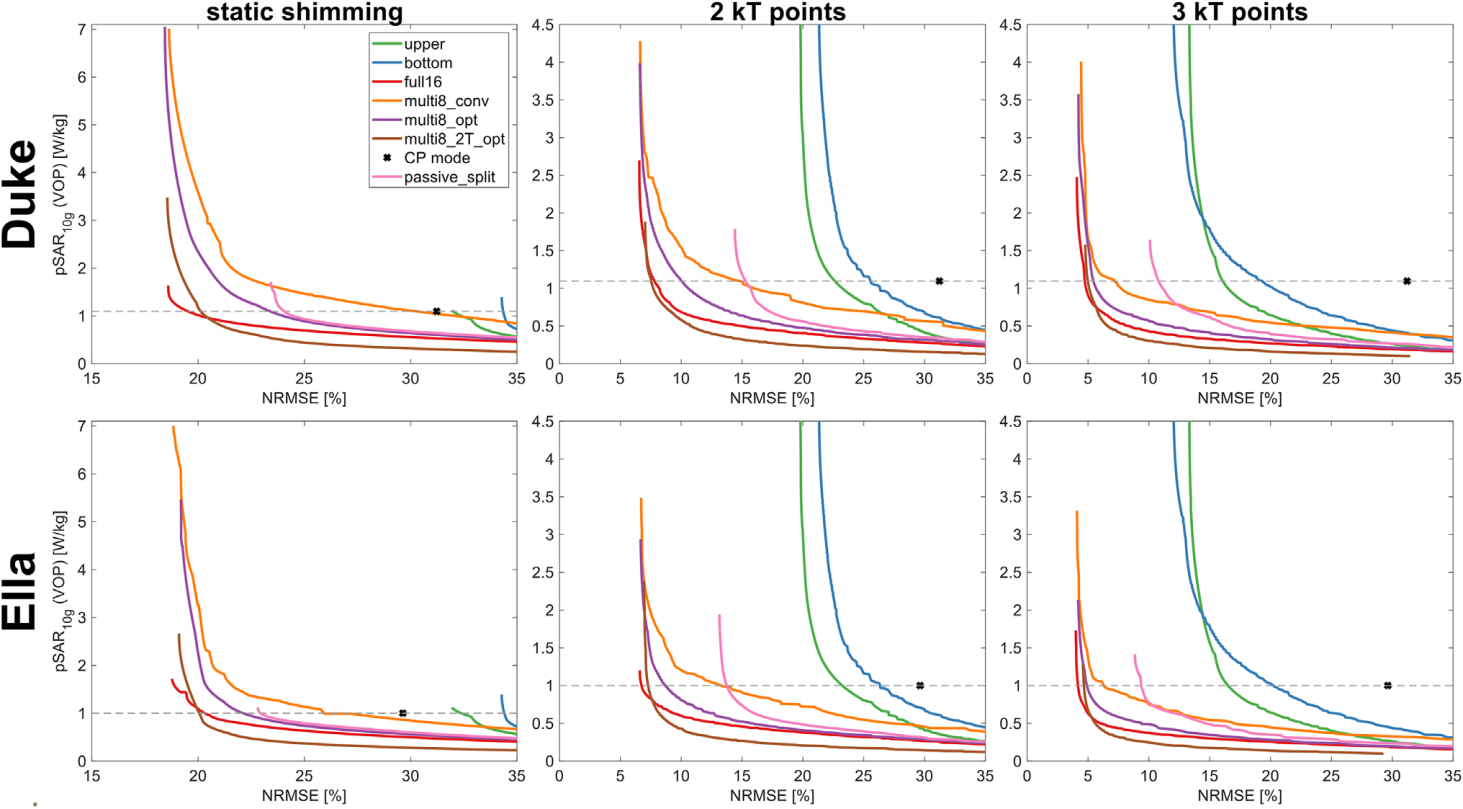
L‐curves obtained from numerical simulations of the Duke and Ella voxel model, showing the trade‐off between excitation homogeneity (NRMSE) and pSAR_10g_ (predicted by the respective VOP‐matrices) for the static and multiplexed driving scenarios described in the Methods section. The black cross indicates the performance of the CP mode in simultaneous 16ch transmission. The intersection of the dashed gray line and the L‐curves marks the respective solution with the same pSAR_10g_ as the CP mode.

Figure 5 shows the L‐curves obtained from simulations of the Duke and Ella voxel models, comparing the achievable tradeoffs between NRMSE and pSAR_10g_ for all driving and pulse design scenarios. Simultaneous transmission on all 16 channels (“full16”) yields a minimum NRMSE of 19%, 7%, and 4% for static RF shimming, 2 kT points, and 3 kT points, respectively (less than 1 percentage point difference between the voxel models). Using only a single row in isolation (“top” and “bottom” scenarios) results in clearly inferior excitation performance. All multiplexed scenarios achieve a similar minimum NRMSE as “full16” (less than 1 percentage point difference to the corresponding “full16” case). The exact equality of the excitation patterns for simultaneous and multiplexed transmission (Equations (2) and (5)), which would imply identical NRMSE values, does not strictly hold here, since the phase evolution due to off‐resonance is taken into account in these calculations. In addition, the RF pulse weights and k‐space locations for the “multi8_opt” and “multi8_2T_opt” scenarios are individually optimized and thus may differ from the corresponding “full16” cases. Simply rearranging the optimized “full16” pulses for multiplexing (i.e., the “multi8_conv” scenario) works well in terms of NRMSE, but results in significantly increased pSAR_10g_ compared to “full16”. This is because the corresponding optimizations were SAR‐constrained according to the “full16” scenario (using $Q_{\text{full}}^{\mathrm{VOP}}$), but the rearranged solutions were evaluated for a multiplexed scenario ($Q_{\text{combined}}^{\mathrm{VOP}}$). In contrast, a dedicated optimization based on $Q_{\text{combined}}^{\mathrm{VOP}}$ (i.e., the “multi8_opt” scenario) yields consistently lower pSAR_10g_ than “multi8_conv” for the same NRMSE. Doubled pulse duration for multiplexing (“multi8_2T_opt”) results in about a 2‐fold reduction of pSAR_10g_ at fixed NRMSE compared to “multi8_opt”. The “passive_split” scenario performs overall worse than the multiplexed scenarios, yielding a minimum NRMSE of [23%, 23%], [14%, 13%], and [10%, 9%] for static RF shimming, 2 kT points, and 3 kT points in [Duke, Ella], respectively.

**FIGURE 6:**
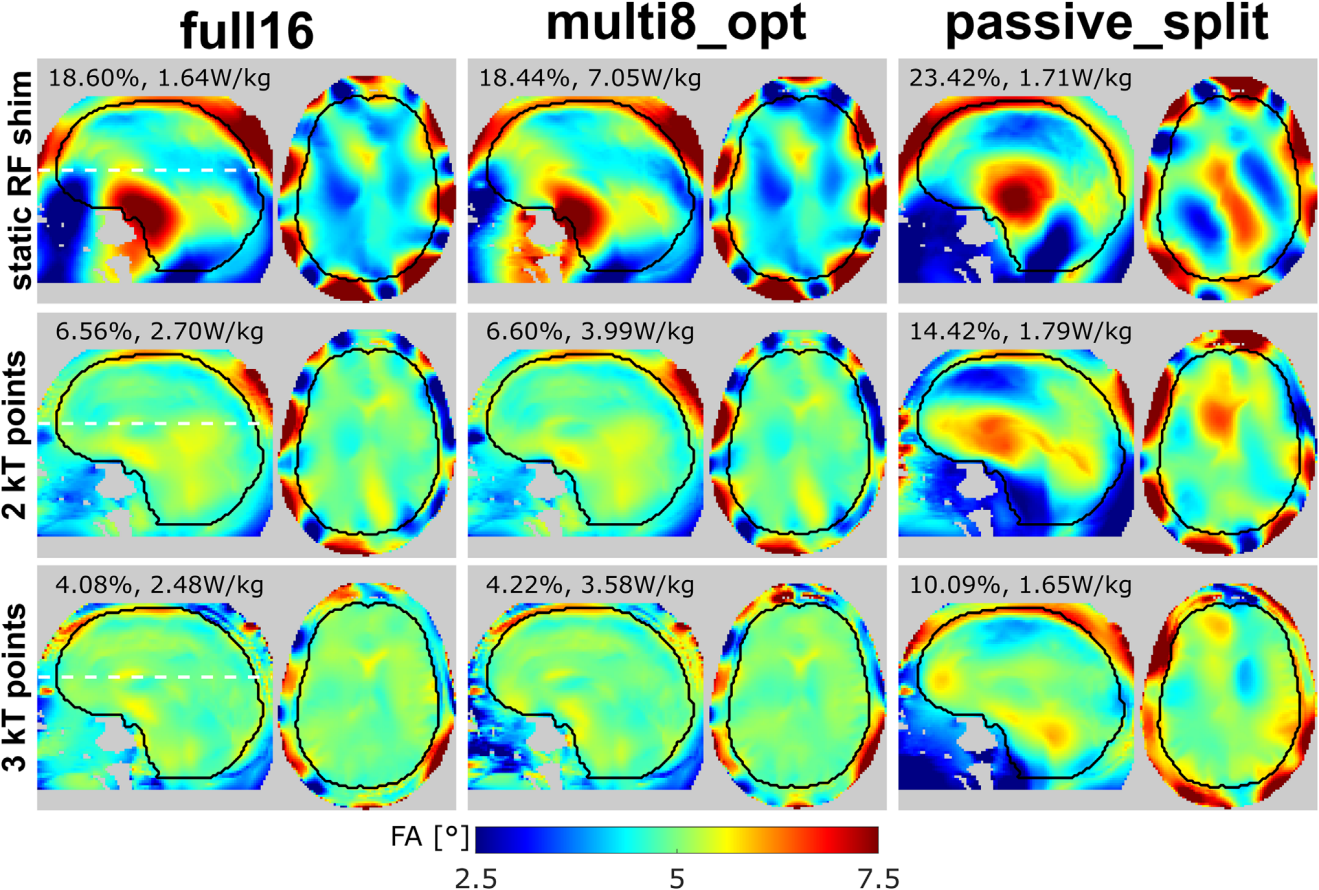
Simulated flip angle maps for the Duke voxel model in the central sagittal and a transversal slice (position indicated by white dashed lines), corresponding to the L‐curves shown in Figure 4. The maps display the pulse with lowest NRMSE, that is, the point at the top left of each L‐curve, respectively. Numbers above the maps indicate the NRMSE and pSAR_10g_ of the respective solution.

**FIGURE 7:**
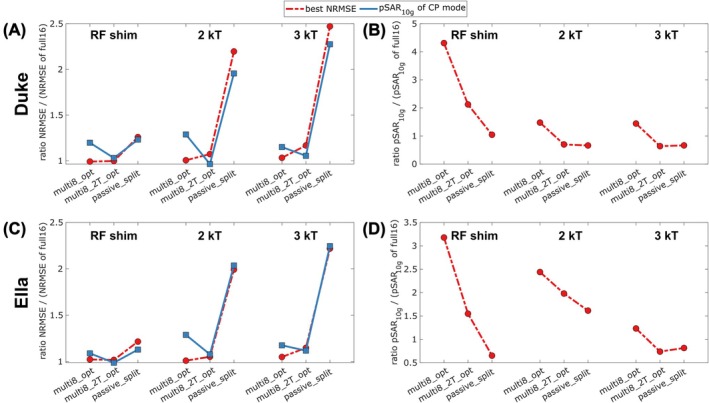
Summarized results of the numerical simulation experiments shown in Figures 5 and 6. Displayed are the relative NRMSE (A, C) and pSAR_10g_ (B, D) normalized to the respective solution of the “full16” scenario, that is, simultaneous transmission on all channels. The solutions with best NRMSE are indicated with red markers, corresponding to the top left corner of the L‐curves in Figure 5, and the solutions with same pSAR_10g_ as the CP mode with blue markers, corresponding to the intersection of the gray lines and the L‐curves in Figure 5.

Figure 6 shows flip angle maps of the respective best solution obtained for simulations of the “full16,” “multi8_opt,” and “passive_split” scenarios. For these unconstrained solutions, multiplexing reduces the NRMSE compared to “passive_split” by factors of [1.27, 2.18, 2.39], while causing a [4.12, 2.23, 2.17]‐fold higher pSAR_10g_ for [static RF shimming, 2 kT points, 3 kT points]. While the absolute pSAR_10g_ values obtained for these unconstrained solutions may still be acceptable for low flip angle imaging situations, they may be problematic for protocols operating close to the local SAR limit. Therefore, as another operating point, the pulse design solutions that yield the same pSAR_10g_ as a “full16” CP mode excitation (Δt=0.2ms) were considered, that is, the intersections of the L‐curves and gray dashed lines in Figure 5. Figure 7 compares the solutions at this operating point with the “best NRMSE” operating point in terms of relative NRMSE and relative pSAR_10g_ (normalized to the respective “full16” value, such that the value 1 corresponds to the ideal case of achieving the same performance as “full16”). When pSAR_10g_ is constrained in this way, “multi8_opt” cannot achieve the same NRMSE as “full16” but suffers from a certain NRMSE penalty (relative NRMSE of [1.20, 1.29, 1.15] for [static RF shimming, 2 kT points, 3 kT points] in Duke, Figure 7A). Consequently, at this operation point, the advantage of “multi8_opt” over “passive_split” in terms of relative NRMSE is small for static RF shimming (1.20 vs. 1.23 for Duke, and 1.09 vs. 1.13 for Ella). However, for 2 kT and 3 kT points excitation, the advantage of “multi8_opt” over “passive_split” is significant (e.g., relative NRMSE 1.15 vs. 2.28 for 3 kT points in Duke). The “multi8_2T_opt” scenario achieves a relative NRMSE close to 1 at both operating points, because the SAR constraint is less binding when the pulse duration is doubled.

**FIGURE 8:**
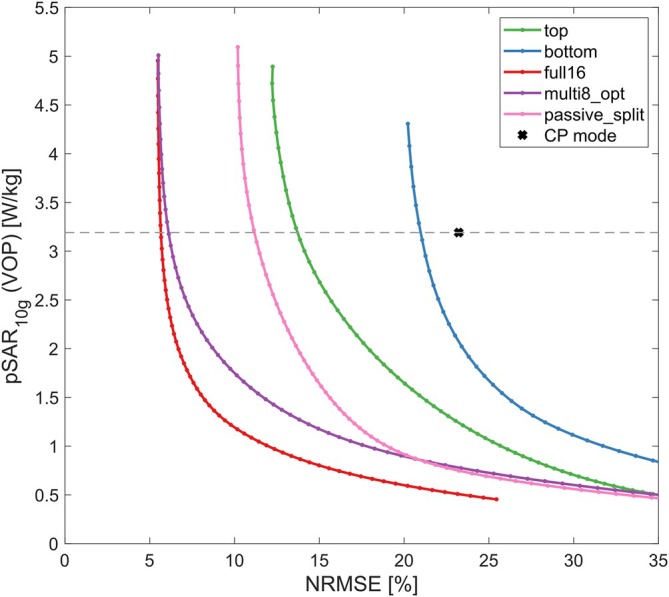
L‐curves for a 3 kT point excitation based on measured single‐channel *B*_1_^+^ maps of a volunteer for the static and multiplexed driving scenarios described in the Methods section. The intersection of the dashed gray line and the L‐curves indicates solutions with the same pSAR_10g_ as the CP mode.

**FIGURE 9:**
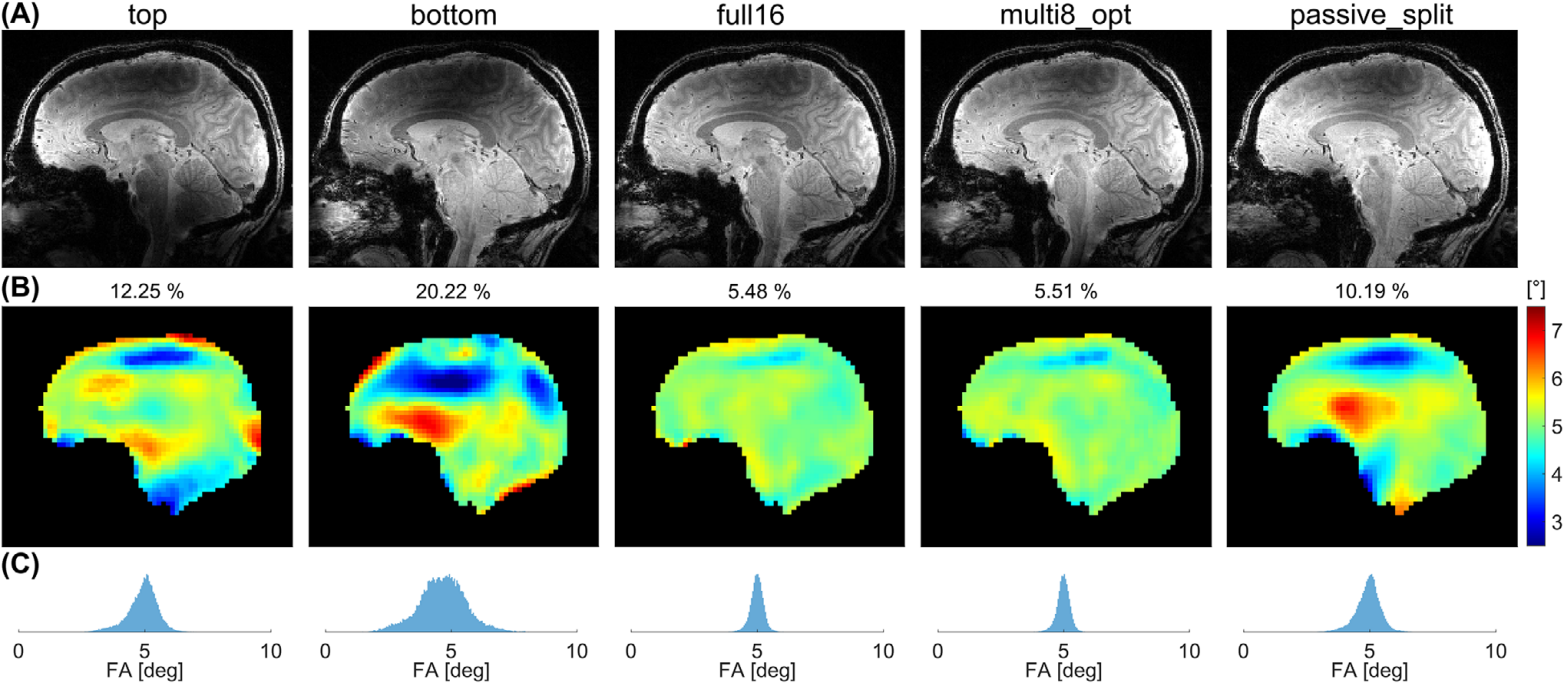
In vivo measurement results: (A) 3D GRE images, (B) flip angle maps (target: 5°), and (C) flip angle histograms across the entire brain volume for the optimized 3 kT points excitation pulses corresponding to the results shown in Figure 8. Displayed are the solutions with the lowest NRMSE (labeled above the flip angle maps) for each driving scenario.

Figure 8 displays the L‐curves Obtained for 3 kT points optimizations based on the measured field maps of the healthy volunteer. Since none of obtained solutions exceeded the local SAR limits, the respective solutions with lowest NRMSE were selected for GRE imaging (Figure 9A). As seen previously in simulations, transmitting only on a single row (“top”/“bottom”) yields inferior flip angle homogeneity. Multiplexing (“multi8_opt”) improves the NRMSE by 2.22‐fold compared to “top” excitation and by 1.85‐fold compared to “passive_split” excitation, achieving almost identical homogeneity as 16ch excitation (Figure 9B,C), while inducing comparable pSAR_10g_. If pSAR_10g_ is constrained to that of the CP mode, the NRMSE of “multi8_opt” is 1.10 times larger than that of “full16” and 1.84 times smaller than that of “passive_split” (Figure 8).

## 5 Discussion

In this work, we have investigated a time‐division multiplexing approach to drive a double row pTx coil array with 16 Tx elements using only 8 RFPAs and SPDT PIN diode switches. With this approach and an appropriate pulse design procedure, almost the same excitation homogeneity can be achieved as with simultaneous transmission on all 16 Tx elements using 16 RFPAs, the latter being only available on a few human UHF scanners. Although the double‐row dipole coil array was designed for high‐resolution anatomical and functional neuroimaging applications, the proposed multiplexing concept is general and independent of a particular coil array design. For example, it could also be applied to combined head and spine imaging at UHF [53, 54], or, speculatively, even for body imaging.

As an inevitable trade‐off, multiplexing comes at the cost of either increased SAR or longer RF pulses compared to simultaneous transmission. This bears analogy to time‐division multiplexing used for signal reception [55, 56, 57, 58]: If the signal from two receiver (Rx) coils is alternatingly fed to a single Rx channel and separated again in post‐processing to restore the information from the independent coils, this results in a $\sqrt{2}$‐times lower SNR than simultaneous reception using two Rx channels, because the total observation time of each coil's signal is halved. Alternatively, doubling the total observation time would allow maintaining the same SNR.

For the proposed multiplexing approach in the small tip angle regime, it is in principle sufficient to first use existing pulse design frameworks designed for conventional non‐multiplexed excitation with all 16 channels, and then to re‐arrange the obtained solution into an 8ch multiplexing scenario according to Equations ((3), (4), (5)). However, as demonstrated by the corresponding “multi8_conv” scenario, this leads to an excessive SAR amplification, because such a pulse design is ignorant of the real electric field interference during multiplexing. Therefore, we have developed a dedicated pulse design method that explicitly takes the multiplexing situation into account (Equations (9) and (10)). This allows controlling the multiplexing‐induced SAR amplification factor and, in most cases, limit it to well below the value of 2 expected for naïve “multi8_conv” re‐arrangement (e.g., 1.44 and 1.23 for 3 kT points, “multi8_opt” in Duke and Ella, see Figure 7B,D) while keeping the total pulse duration constant. For simplicity, the pulse design approach considered here constrains only the peak local SAR, since this is usually the most binding constraint for UHF head imaging [41] and no exceedance of RF power or global SAR limits was retrospectively observed for the obtained pulses. Adding RF power and global SAR constraints to the optimization problem would be straightforward.

In terms of the pulse design workflow, the multiplexing approach requires the same calibration time as the “full16” scenario, because it relies on repeating an 8ch *B*_1_^+^ mapping sequence for both rows of the array. While the experiments shown in this work were using tailored RF pulses that were optimized on‐the‐fly during the scan session, the multiplexing approach is fully amenable to the concept of universal pulses [59] if a database of *B*_1_^+^ maps is available.

The current implementation of VOP‐based SAR monitoring at the scanner system cannot take into account the temporal degree of freedom introduced by multiplexing. Therefore, a cornerstone of the proposed method is the joint compression of the Q‐matrices associated with the rows of the array into a single set of VOPs (Figure 3A). This guarantees safe operation of the coil in all scenarios, that is, never underestimating the actual physical SAR. Regarding the overestimation by the VOPs compared to voxel‐wise Q‐matrices (Figure 3D), there are two counteracting effects: On the one hand, $Q_{\text{combined}}^{\mathrm{VOP}}$ has to jointly represent two very different electric field patterns, that is, those caused by the upper and lower row, which is expected to a lead to a large overestimation. On the other hand, $Q_{\text{combined}}^{\mathrm{VOP}}$ represents an 8ch system, while $Q_{\text{full}}^{\mathrm{VOP}}$ represents a 16ch system. The latter is known to require a larger number of VOPs for fixed overestimation, or, conversely, to cause higher overestimation when the number of VOPs is fixed [60]. In the present case, both effects appear to be largely balanced, allowing for viable pulse design solutions using $Q_{\text{combined}}^{\mathrm{VOP}}$. Nevertheless, the effect of different VOP compression strategies [61] and the potential benefit of smaller VOP‐matrices enabled by multiplexing for high element count arrays warrant further investigation. As a speculative outlook, an implementation of multiplexing‐aware online SAR monitoring that alternates between different sets of VOP files and thus causes even less overestimation would at least be technically conceivable.

The advantage of multiplexing for simple static RF shimming was found to be limited when compared to the “passive_split” scenario. This is not surprising, since theoretically any static RF shim could be realized with a single RFPA, a splitter, and appropriate attenuators and phase shifters. In contrast, kT points pulses were found to benefit significantly from the independent degrees of freedom provided by 16 static or 8 multiplexed RFPAs, respectively. In particular, for static shimming, the L‐curves in Figure 5 corresponding to the multiplexing scenarios reach considerably higher pSAR_10g_ values than for the kT points. We attribute this to the numeric conditioning of the respective design matrices: For static shimming, the only spatial basis functions are the transmit profiles, which overlap between the rows, requiring large pulse amplitudes to achieve the interference patterns required for flip angle homogenization. In contrast, the gradient blips of the kT points pulses add plane waves to the spatial basis functions, which offer significantly more degrees of freedom for achieving homogenizing interferences while keeping pulse amplitudes low.

The proposed approach of time‐interleaved excitation through the two rows of the array has similarity with the TIAMO method [62, 63], in which two RF shim modes are alternated between the repetitions of the pulse sequence. In fact, the RF switch of the present work could be used for a simple TIAMO application, alternating between the two rows on a repetition‐by‐repetition level instead of the proposed sub‐pulse level. However, the primary goal of the present work was to investigate the potential of multiplexing to retain as much flexibility and performance of the full 16 Tx channel system as possible, which requires fast switching on the sub‐pulse duration time scale.

As an alternative approach to driving a larger number of Tx elements with a smaller number of RFPAs, array‐compressed pTx [24] has been proposed. In this approach, a larger number of coils are connected to a smaller number of channels via an array compression network consisting of power splitters, attenuators, and phase shifters [25, 64, 65, 66]. The compression weights that need to be represented by these components are jointly optimized with the pulse waveforms. Similar to this method, time‐division multiplexing requires additional hardware in the form of the RF switch, as well as dedicated pulse design algorithms. Both methods have their advantages and disadvantages due to their inherently different approaches of preserving as many of the degrees of freedom of the ideal one‐channel‐per‐coil situation as possible: For array‐compressed pTx, some of these degrees of freedom are cast into hardware in the form of the compression network. This adds considerable complexity to practical implementations, thus, the necessary components are an area of active research [64, 65, 66] and have so far only been demonstrated for a 2‐channel, 8‐coil setup [25]. In contrast, time‐division multiplexing distributes the degrees of freedom in time, which can be realized by much simpler hardware that is not dependent on the particular coil, subject, or pulse design scenario. However, it comes at the expense of the demonstrated trade‐off between increased SAR or pulse duration. In conclusion, the proposed multiplexing approach provides a complementary experimental tool to cope with the limited number of RFPAs available for UHF pTx applications with state‐of‐the‐art coil arrays.

Future directions will be to investigate the multiplexing concept for other pulse design objectives, such as slab‐selective or inner‐volume excitation [67], as well as investigating the approach at different field strengths. In addition, it may be of interest to explore different coil designs (e.g., loop and dipole arrays) and their suitability for multiplexing, depending on the assignment of coil elements to the switchable groups. For example, alternating between loop and dipole elements could provide performance benefits for pTx even if not all elements could be driven simultaneously.

## 6 Conclusion

Time‐division multiplexing offers novel ways to flexibly utilize advanced pTx coil arrays with high element counts in systems with a limited number of RFPAs. In numerical simulations and measurements on a 9.4 T human scanner, similar excitation homogeneity could be achieved for 8ch multiplexed and 16ch simultaneous transmission, but at the cost of either increased local SAR or pulse duration.

## Funding

This work was supported by European Research Council (834940), Max‐Planck‐Gesellschaft, and Deutsche Forschungsgemeinschaft (530130666).

## Supporting information


**Data S1:** Supporting Information.

## Data Availability

Data and code for demonstration of the methods proposed in this study are openly available at https://github.com/fglang/tdm_ptx.
