# Supplementary material for: Time‐Division Multiplexing for Parallel Transmission at Ultra‐High Field With Limited RF Channels
Source: Magn Reson Med. 2025 Dec 19;95(5):2580–93. doi: 10.1002/mrm.70230 (PMC12962223; doi:10.1002/mrm.70230)
Supplement: Supplementary file 1 — Data S1: Supporting Information. [file MRM-95-2580-s001.docx]

***Supporting Information***

Time-Division Multiplexing for Parallel Transmission at Ultra-High Field with Limited RF Channels

**Felix Glang^1,2*^, Georgiy A. Solomakha^1^, Dario Bosch^1,3,4^, Klaus Scheffler^1,3^, Nikolai I. Avdievich^1^**

^1^Magnetic Resonance Center, Max Planck Institute for Biological Cybernetics, Tübingen, Germany

^2^Institute of Biomedical Imaging, Graz University of Technology, Graz, Austria

^3^Department of Biomedical Magnetic Resonance, Eberhard Karls University Tübingen, Tübingen, Germany

^4^MRI Core Facility of the Medical Faculty, University of Tübingen, Otfried-Müller-Straße 51, Tübingen, 72076, Germany.

# SAR amplification due to multiplexing

In the following, we investigate the effect of the proposed multiplexing method on the local SAR, comparing it to simultaneous transmission. The time-averaged SAR in a certain spatial location caused by an RF pulse of $N_{t}$ waveform samples of duration $\Delta t$ averaged over the repetition time $\mathrm{TR}$ can be expressed as^1,2^

| $\mathrm{SAR}=\frac{1}{\mathrm{TR}} \sum_{\tau=1}^{N_{t}} \frac{\sigma}{2\rho}\left\vert\left\vert\mathcal{E}_{\tau} \right\vert\right\vert^{2}\Delta t$ | [S1] |
| --- | --- |

with the tissue conductivity $\sigma$, mass density $\rho$, and the total electric field at each time step $\mathcal{E}_{\tau}$.

Consider the contribution of a single time step ($N_{t}=1$) when driving all coils from both group A and group B simultaneously, creating the total electric field $\mathcal{E}=\mathcal{E}_{A}+\mathcal{E}_{B}\in\mathbb{C}^{3}$. Ignoring the dependence on $\mathrm{TR}$, $\sigma$, and $\rho$, which are kept constant throughout the following comparison, one obtains

| $\mathrm{SA}R_{\mathrm{simult}} \sim\left\vert\left\vert\mathcal{E}_{A}+\mathcal{E}_{B} \right\vert\right\vert^{2}\cdot\Delta t=\left( \left\vert\left\vert\mathcal{E}_{A} \right\vert\right\vert^{2}+\left\vert\left\vert\mathcal{E}_{B} \right\vert\right\vert^{2}+2\cdot\mathfrak{N}\left( \mathcal{E}_{A}^{H}\mathcal{E}_{B} \right) \right)\cdot\Delta t$ | [S2] |
| --- | --- |

Note that the third summand describes the interference between the field components of the two coil groups.

In case of multiplexed transmission, i.e., first pulsing coil group A and then coil group B for the time $\Delta t/2$ and with doubled amplitudes, respectively, one obtains

| $\mathrm{SAR}_{\mathrm{multiplex}} \sim\left\vert\left\vert2\cdot\mathcal{E}_{A} \right\vert\right\vert^{2}\cdot\frac{\Delta t}{2}+\left\vert\left\vert2\cdot\mathcal{E}_{B} \right\vert\right\vert^{2}\cdot\frac{\Delta t}{2}=2\cdot\left( \left\vert\left\vert\mathcal{E}_{A} \right\vert\right\vert^{2}+\left\vert\left\vert\mathcal{E}_{B} \right\vert\right\vert^{2} \right)\cdot\Delta t$ | [S3] |
| --- | --- |

where no interference term of A and B occurs due to the sequential transmission.

The ratio of the two SAR cases described above, Eq. S2 and Eq. S3, is

| $\xi\equiv\frac{\mathrm{SA}R_{\mathrm{simult}}}{\mathrm{SAR}_{\mathrm{multiplex}}}=\frac{1}{2}+\frac{\mathfrak{N}\left( \mathcal{E}_{A}^{H}\mathcal{E}_{B} \right)}{\left\vert\left\vert\mathcal{E}_{A} \right\vert\right\vert^{2}+\left\vert\left\vert\mathcal{E}_{B} \right\vert\right\vert^{2}}=\frac{1}{2}+\frac{R}{R^{2}+1}\cdot\cos(\psi)$ | [S4] |
| --- | --- |

with the amplitude ratio $R=\frac{\left| \left| \mathcal{E}_{A} \right| \right|}{\left| \left| \mathcal{E}_{B} \right| \right|}$ and the angle $\psi=\angle(\mathcal{E}_{A},\mathcal{E}_{B})$. Note that this angle defined by $\cos\left( \psi\right)=\frac{\mathfrak{N}\left( \mathcal{E}_{A}^{H}\mathcal{E}_{B} \right)}{\left| \left| \mathcal{E}_{A} \right| \right|\cdot\left| \left| \mathcal{E}_{B} \right| \right|}$ takes both the spatial orientation and the phase relationship between the superimposed electric fields into account. The ratio $\xi$ can be interpreted as the inverse SAR penalty factor incurred by multiplexing. It takes values between 0 and 1, meaning that $\mathrm{SAR}_{\mathrm{multiplex}}$ can never be smaller than $\mathrm{SA}R_{\mathrm{simult}}$.

The following special cases can be identified:

- Fully constructive interference: $\mathcal{E}_{A}$ and $\mathcal{E}_{B}$ of same amplitude ($R=1)$ and $\psi=0^{\circ}$. This leads to $\xi=1$, i.e., identical SAR values for both cases, no SAR penalty due to multiplexing
- Fully destructive interference: $R=1$ and $\psi=180^{\circ}$. This leads to $\xi=0$, i.e., no SAR in the simultaneous transmission case due to perfect cancellation of the electric fields, infinite SAR penalty due to multiplexing
- Disjoint electric field distributions: $R=0$ or $R\to\infty$ ($\left| \left| \mathcal{E}_{A} \right| \right|\gg\left| \left| \mathcal{E}_{B} \right| \right|$ or $\left| \left| \mathcal{E}_{A} \right| \right|\ll\left| \left| \mathcal{E}_{B} \right| \right|$), or orthogonal fields, $\psi=\pm90^{\circ}$. Both lead to $\xi=1/2$, i.e., two-fold increase of SAR due to multiplexing.

Perfect constructive or destructive interference are very unlikely to occur in reality, especially taking into account that for realistic SAR computations, the electric fields are commonly averaged over 10g regions. Assuming an equal probability for all possible values of $R$ and $\psi$, the expected value of the SAR penalty factor can be calculated as

| $\mathbb{E}\left( \xi\right)=\lim_{R\to\infty}\frac{1}{R}\int_{0}^{R} dR'\frac{1}{2\pi}\int_{0}^{2\pi} d\psi\xi\left( R^{'},\psi\right)=\frac{1}{2}$ | [S5] |
| --- | --- |

Thus, similar to the average pulse power in Eq. 8 in the main text, the SAR amplification due to multiplexing at a fixed spatial location is expected to be usually around 2.

Note that all these comparisons hold on a voxel-by-voxel level and therefore not for the peak local SAR. Since the peak local SAR might occur in different locations (“hot spots”) for coil groups A and B driven sequentially, the peak local SAR accumulated over a multiplexed pulse can actually be lower than for simultaneous transmission, for which the “hotspot” remains at the same location.

# Simulation results for the CP mode in the Ella voxel model

**
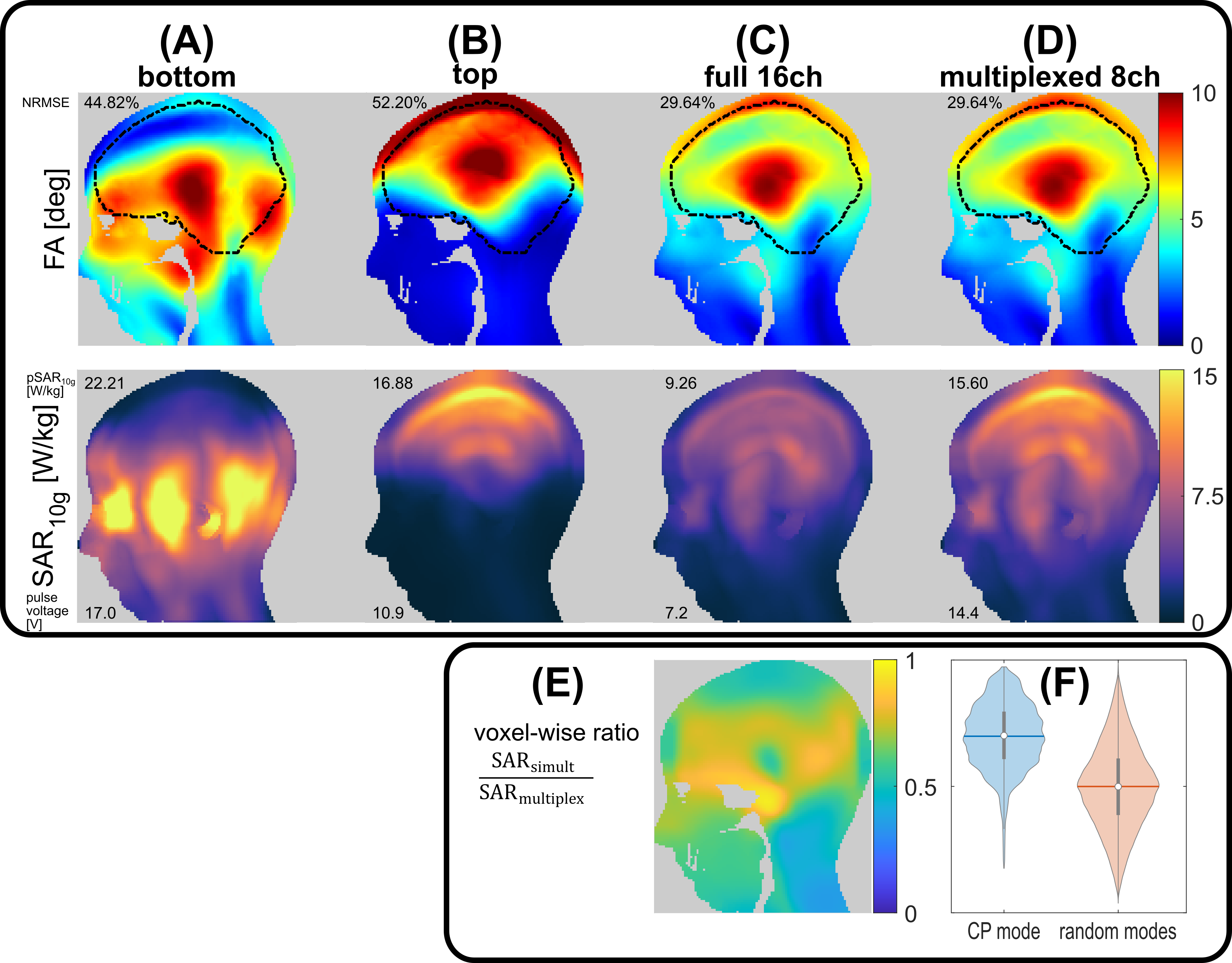
**

**Supporting Information Figure S1.** Simulated CP mode excitation in the Ella voxel model for **(A, B)** 8ch single-row transmission, **(C)** simultaneous 16ch transmission using all elements, and **(D)** time-division multiplexed 8ch transmission. For each case, the same total pulse duration was assumed and the pulse amplitude was scaled to achieve an average flip angle of 5° in the outlined brain mask. The SAR_10g_ maps are maximum intensity projections across the left-right direction. Numbers indicate the normalized root-mean-squared error (NRMSE) with respect to 5° excitation (first row), pSAR_10g_ (second row top, assuming 100% RF duty cycle), and obtained pulse voltages (second row, bottom). **(E)** Voxel-wise ratio of the SAR_10g_ maps in (C) and (D), showing the inverse SAR amplification caused by multiplexing (displayed in a single sagittal slice). **(F)** Violin plots of the same voxel-wise SAR ratio for the CP mode (left violin, corresponding to the map in (E)), as well as for the same 4096 random RF modes as shown in Figure 3 (right violin). Within the violins, the white dot indicates the median, the horizontal lines the mean, and the gray bars the interquartile range of the respective distribution.

# Analytical Jacobians for the pulse design problem

In the following, we summarize the sources, considerations and final expressions for implementing analytical Jacobians to solve the pulse design problem stated in Eq. 9 in the main text.

The objective function for the magnitude least-squares pulse design problem is

$$\mathcal{L=}\left| \left| \left| \mathbf{A}\boldsymbol{P} \right|-\boldsymbol{b} \right| \right|_{2}^{2}$$

with the design matrix $\mathbf{A}$ of shape $N_{r}\times N_{t}\cdot N_{c}$, the vectorized pulse samples $\boldsymbol{P}$ of shape $N_{t}\cdot N_{c}$ and the vector of target flip angles $\boldsymbol{b}$ of shape $N_{r}$. Following the Appendix of Guerin et al.^3^, Eqs. A1-A5, the relevant derivatives of $\mathcal{L}$ with respect to the real and imaginary part of $\boldsymbol{P}$ are given by

$$\frac{\partial\mathcal{L}}{\partial\mathfrak{N(}\boldsymbol{P})}=2\mathfrak{N}\left\{ \boldsymbol{A}^{H}\left( \left( \left| \boldsymbol{AP} \right|-\boldsymbol{b} \right)\odot e^{i\angle\left( \boldsymbol{AP} \right)} \right) \right\}$$

$$\frac{\partial\mathcal{L}}{\partial\mathfrak{I(}\boldsymbol{P})}=2\mathfrak{I}\left\{ \boldsymbol{A}^{H}\left( \left( \left| \boldsymbol{AP} \right|-\boldsymbol{b} \right)\odot e^{i\angle\left( \boldsymbol{AP} \right)} \right) \right\}$$

where $\odot$ denotes the element-wise product and $\angle(\cdot)$ the element-wise argument of a complex vector.

The derivatives of $\mathcal{L}$ with respect to the k-space locations can be found following Yip et al.^4^ (Eqs. 18-19), who derived corresponding expressions for an ordinary least-squares objective, in combination with the derivatives for the magnitude-least squares objective employed here. One obtains

$$\frac{\partial\mathcal{L}}{\partial k_{j}^{(\rho)}}=2\mathfrak{I}\left\{ \sum_{i=1}^{N_{r}} \left[ \left( \left| \boldsymbol{AP} \right|-\boldsymbol{b} \right)\odot e^{i\angle\left( \boldsymbol{AP} \right)}\odot\boldsymbol{r}^{(\rho)} \right]_{i}\sum_{c=1}^{N_{c}} A_{i,\left( jc \right)}^{*}P_{\left( jc \right)}^{*} \right\}$$

for all spatial directions $\rho=\{x,y,z\}$ and time steps $j=1,\ldots,N_{t}$.

For the SAR constraints, following Hoyos-Idrobo et al.^5^, the constraint function for a single VOP $\boldsymbol{Q}_{n}^{\mathrm{VOP}}$ can be written as

$$\mathcal{C}_{n}=\frac{\Delta t}{\mathrm{TR}}{\cdot\boldsymbol{P}}^{H}\left( \boldsymbol{Q}_{n}^{\mathrm{VOP}}\otimes\boldsymbol{I}_{N_{t}} \right)\boldsymbol{P}\boldsymbol{-}l\mathrm{SA}R_{\max}$$

with the $N_{t}\times N_{t}$ identity matrix $\boldsymbol{I}_{N_{t}}$ and the Kronecker product $\otimes$. Note that in contrast to Hoyos-Idrobo et al.^5^, we sort $\boldsymbol{P}$ first by time and then by channel, which explains the swapped Kronecker product order compared to their expression. The relevant derivatives of this quadratic form are

$$\frac{\partial\mathcal{C}_{n}}{\partial\mathfrak{N(}\boldsymbol{P})}=\frac{\Delta t}{\mathrm{TR}}\cdot2\mathfrak{N}\left\{ \left( \boldsymbol{Q}_{n}^{\mathrm{VOP}}\otimes\boldsymbol{I}_{N_{t}} \right)\boldsymbol{P} \right\}$$

$$\frac{\partial\mathcal{C}_{n}}{\partial\mathfrak{I(}\boldsymbol{P})}=\frac{\Delta t}{\mathrm{TR}}\cdot2\mathfrak{I}\left\{ \left( \boldsymbol{Q}_{n}^{\mathrm{VOP}}\otimes\boldsymbol{I}_{N_{t}} \right)\boldsymbol{P} \right\}$$

which can be efficiently vectorized over the entire set of VOP matrices.

# References

1. Graesslin I, Homann H, Biederer S, et al. A specific absorption rate prediction concept for parallel transmission MR. *Magnetic Resonance in Medicine*. 2012;68(5):1664-1674. doi:10.1002/mrm.24138

2. IEC 60601-2-33. *Medical Electrical Equipment – Part 2-33: Particular Requirements for the Basic Safety and Essential Performance of Magnetic Resonance Equipment for Medical Diagnosis, Edition 3.1*. Geneva: International Electrotechnical Commission; 2013.

3. Guérin B, Stockmann JP, Baboli M, Torrado-Carvajal A, Stenger AV, Wald LL. Robust time-shifted spoke pulse design in the presence of large B0 variations with simultaneous reduction of through-plane dephasing, B1+ effects, and the specific absorption rate using parallel transmission. *Magnetic Resonance in Medicine*. 2016;76(2):540-554. doi:10.1002/mrm.25902

4. Yip CY, Grissom WA, Fessler JA, Noll DC. Joint design of trajectory and RF pulses for parallel excitation. *Magnetic Resonance in Medicine*. 2007;58(3):598-604. doi:10.1002/mrm.21262

5. Hoyos-Idrobo A, Weiss P, Massire A, Amadon A, Boulant N. On Variant Strategies to Solve the Magnitude Least Squares Optimization Problem in Parallel Transmission Pulse Design and Under Strict SAR and Power Constraints. *IEEE Transactions on Medical Imaging*. 2014;33(3):739-748. doi:10.1109/TMI.2013.2295465
